# Clinical impact of aortic valve replacement in patients with moderate mixed aortic valve disease

**DOI:** 10.3389/fcvm.2023.1259188

**Published:** 2023-08-23

**Authors:** Hirokazu Onishi, Masaki Izumo, Toru Ouchi, Haruhito Yuki, Toru Naganuma, Tatsuya Nakao, Sunao Nakamura

**Affiliations:** ^1^ Department of Cardiology, New Tokyo Hospital, Chiba, Japan; ^2^ Department of Cardiology, St. Marianna University School of Medicine, Kanagawa, Japan; ^3^ Department of Cardiovascular Surgery, New Tokyo Hospital, Chiba, Japan

**Keywords:** aortic regurgitation, aortic stenosis, aortic valve replacement, heart failure, mixed aortic valve disease

## Abstract

**Background:**

Information is scarce regarding the clinical implications of aortic valve replacement (AVR) for patients suffering from moderate mixed aortic valve disease (MAVD), characterized by a combination of moderate aortic stenosis (AS) and regurgitation (AR). The objective of this retrospective study was to explore the clinical effects of AVR in individuals with moderate MAVD.

**Methods:**

We examined the clinical data from patients with moderate MAVD and preserved left ventricular ejection fraction, who had undergone echocardiography in the period spanning from 2010 to 2018. Moderate AS was defined as aortic valve area index of 0.60–0.85 cm^2^/m^2^ and peak velocity of 3.0–4.0 m/s. Moderate AR was defined as a vena contracta width of 3.0–6.0 mm. The primary endpoint was a composite of all-cause death and heart failure hospitalization.

**Results:**

Among 88 patients (mean age, 74.4 ± 6.8 years; 48.9%, men), 44 (50.0%) required AVR during a median follow-up period of 3.3 years (interquartile range, 0.5–4.9). Mean values of specific aortic valve variables are as follows: aortic valve area index, 0.64 ± 0.04 cm^2^/m^2^; peak velocity, 3.40 ± 0.30 m/s; and vena contracta width, 4.1 ± 0.7 mm. The primary endpoint occurred in 32 (36.4%) patients during a median follow-up duration of 5.3 years (interquartile range, 3.2–8.0). Multivariable analysis revealed that AVR was significantly associated with the endpoint (hazard ratio, 0.248; 95% confidence interval, 0.107–0.579; *p* = 0.001) after adjusting for age, B-type natriuretic peptide, and the Charlson comorbidity index. Patients who underwent AVR during follow-up had significantly lower incidence rates of the endpoint than those managed with medical treatment (10.2% vs. 44.1% at 5 years; *p* < 0.001).

**Conclusions:**

Approximately half of the patients diagnosed with moderate MAVD eventually necessitated AVR throughout the period of observation, leading to positive clinical results. Vigilant tracking of these patients and watchful monitoring for signs requiring AVR during this time frame are essential.

## Introduction

Mixed aortic valve disease (MAVD), characterized by aortic stenosis (AS) with regurgitation (AR), places an elevated overall strain on the left ventricle in comparison to either isolated AS or AR, as it brings together both pressure and volume loads. As a result, it's anticipated that patients with moderate-to-severe MAVD, described as moderate-to-severe AS coexisting with moderate-to-severe AR, would experience more adverse clinical outcomes than those only having moderate-to-severe AS (1). According to the current guidelines, aortic valve replacement (AVR) should be considered if symptomatic patients with preserved left ventricular ejection fraction (LVEF) have a peak aortic valve (AV) velocity of ≥4.0 m/s. Further, previous studies showed that AVR available on demand was suggested to significantly reduce the risk of all-cause mortality in such patients (2, 3). Recent research suggests that, while patients with moderate AS have adverse clinical outcomes (4, 5), patients with moderate MAVD, namely, moderate AS combined with moderate AR, run the risk of progressively worsening AV hemodynamics and negative clinical outcomes (6, 7). These outcomes are considerably worse than those observed in patients with either moderate AS or AR individually, but align with those seen in patients exclusively suffering from severe AS (6). Additionally, given that AVR was the most common occurrence among the components of a composite endpoint, which included AVR, deteriorating heart failure, and all-cause mortality (6, 7), it's projected that AVR may be potentially unavoidable. Moreover, the clinical outcomes following AVR could be significant in patients with moderate MAVD; yet, contrasting with moderate-to-severe MAVD, there's a paucity of data regarding the influence of AVR on clinical outcomes in individuals with moderate MAVD. Consequently, the purpose of this study was to scrutinize the effect of AVR on clinical outcomes in patients with moderate MAVD.

## Methods

### Patient population

This investigation involved a retrospective examination of demographic, clinical, and echocardiographic information from patients suffering from moderate MAVD, and with maintained LVEF (LVEF ≥50%). These patients received treatment at our institution during the time period stretching from January 2010 to December 2018. Based on published guidelines (8, 9), moderate AS was defined as an AV area (AVA) index of >0.60 cm^2^/m^2^ to ≤0.85 cm^2^/m^2^ and a peak AV velocity of ≥3.0 to <4.0 m/s. The determination of AR severity employed integrative methods that relied on semi-quantitative indicators such as the vena contracta width and the ratio of AR jet width to left ventricular outflow tract (LVOT) width, as well as qualitative markers, which included pressure half-time and the presence of descending aortic diastolic flow reversal. Moderate AR was semi-quantitatively characterized by a vena contracta width ranging from ≥3.0 mm to <6.0 mm, and a ratio of AR jet width to LVOT width between ≥25% and <65%. The Charlson comorbidity index was calculated for each patient (10). Individuals were disqualified from the study based on the following exclusion parameters: being older than 85 years, having mitral regurgitation or stenosis more severe than moderate as per current standards (9, 11), having previously undergone AVR, possessing a LVEF of less than 50%, suffering from hypertrophic cardiomyopathy, having congenital heart disease (bicuspid AV being the exception), and experiencing LVOT obstruction. Information on patient characteristics, echocardiographic data, and follow-up were collected from medical histories and echocardiography summaries. The research plan adhered to the guidelines established in the Declaration of Helsinki and received approval from the Institutional Review Board of New Tokyo Hospital. The need for informed consent was waived due to the retrospective design of the study.

### Echocardiographic measurements

Echocardiography was conducted using either the Vivid E9 (General Electric Healthcare, Little Chalfont, UK), the iE33 (Philips Healthcare, Andover, MA), or the EPIQ7 systems (Philips Healthcare, Andover, MA), conforming to pertinent guidelines (8, 9, 11, 12). The resultant data were archived on a dedicated workstation for subsequent offline evaluation. Measurements for left ventricular end-diastolic and end-systolic volumes, LVEF, and left atrial volume were taken employing the biplane Simpson disk technique. The software TomTec Arena version 2.40 (TomTec Imaging Systems, Munich, Germany) was used for vendor-independent evaluation of left ventricular global longitudinal strain. Two-dimensional echocardiography was employed for calculations related to the left ventricular mass index and relative wall thickness. Measurements for peak AV velocity, along with peak and mean AV pressure gradients were obtained in the continuous-wave Doppler mode from apical or right parasternal approaches where feasible, and were evaluated using the simplified Bernoulli equation.

### Follow-up and study endpoints

Data from follow-up sessions were gathered through discussions with the patients themselves, their family members, or their respective doctors. Special attention was paid to collecting information pertaining to AVR, instances of heart failure (HF) that required hospitalization, and mortality. Recommendations for AVR were made by cardiologists or cardiac surgeons affiliated with our hospital, with these recommendations guided by the prevailing guidelines (13).

The primary endpoint of the study was established as a combination of all-cause death and HF hospitalization. If a patient was hospitalized for HF at the time of the initial echocardiography, any subsequent event after being discharged from that particular hospitalization was regarded as an incident.

### Statistical analyses

Variables of the categorical variety are displayed as frequencies, and the Chi-squared or Fisher's exact test was employed for their analysis, depending on suitability. Continuous variables are depicted as either mean ± standard deviation or as the median accompanied by the interquartile range (IQR), and the *t*-test or the Wilcoxon rank-sum test was utilized for their comparison, as deemed suitable. The Kaplan–Meier method was used to ascertain the cumulative incidence of the pre-established composite endpoint, with the date of the primary echocardiography serving as the initial time point (*t* = 0). The Holm method was adopted for pairwise comparisons involving multiple comparisons. Both univariate and multivariate Cox proportional hazard regression analyses were carried out to evaluate the connection between AVR and the primary endpoint. The specific variety of AVR executed, whether surgical AVR (SAVR) or transcatheter AVR (TAVR), was incorporated in the multivariate Cox regression analysis, in addition to AVR. A *p*-value of less than 0.05 from univariate analysis was the criterion for choosing variables for multivariate models. To circumvent overfitting, the quantity of variables included in the multivariable models was capped at a maximum of one for every eight events (14, 15). Every statistical test was conducted using two-tailed testing. A *p*-value of less than 0.05 was deemed to be statistically significant in the context of multivariate analysis. The data were analyzed using the SPSS software for Windows, version 25.0 (SPSS Inc, Chicago, Illinois, USA).

## Results

### Patient characteristics

The study scrutinized the records of 88 patients who satisfied the eligibility requirements and had received an initial echocardiography between the period of January 2010 and December 2018 (Table 1). Mean age of the cohort was 74.4 ± 6.8 years and 43 of them (48.9%) were men. New York Heart Association (NYHA) functional class I and II were seen in 60 (68.2%) and 28 (31.8%) patients, respectively. Median BNP level of the cohort was 59.5 pg/ml (IQR, 29.6–105.8) and the Charlson comorbidity index was 5.0 ± 1.9. Echocardiographic data are listed in Table 2. Mean values of specific AV variables are as follows: AVA index, 0.64 ± 0.04 cm^2^/m^2^; peak AV velocity, 3.40 ± 0.30 m/s; vena contracta width, 4.1 ± 0.7 mm; and AR jet width/LVOT width, 35.5% ± 5.6%.

**Table 1:** Patient characteristics.

Variables	All patients (*n* = 88)	Medical treatment (*n* = 44, 50.0%)	AVR (*n* = 44, 50.0%)	*p*-value
Age, years	74.4 ± 6.8	74.6 ± 5.7	74.3 ± 7.9	0.865
Men, *n*	43 (48.9)	23 (52.3)	20 (45.5)	0.670
Body mass index, kg/m^2^	23.3 ± 2.7	23.7 ± 3.1	22.9 ± 2.3	0.195
Hypertension, *n*	58 (65.9)	27 (61.4)	31 (70.5)	0.500
Diabetes mellitus, *n*	14 (15.9)	11 (25.0)	3 (6.8)	0.039
Dyslipidemia, *n*	45 (51.1)	17 (38.6)	28 (63.6)	0.032
Chronic kidney disease, *n*	45 (51.1)	27 (61.4)	18 (40.9)	0.087
Hemodialysis, *n*	7 (8.0)	5 (11.4)	2 (4.5)	0.434
B-type natriuretic peptide, pg/ml (median)	59.5 (29.6–105.8)	64.2 (15.3–807.8)	55.8 (6.1–563.2)	0.395
Atrial fibrillation/flutter, *n*	18 (20.5)	10 (22.7)	8 (18.2)	0.792
Previous myocardial infarction, *n*	10 (11.4)	9 (20.5)	1 (2.3)	0.015
Previous PCI, *n*	29 (33.0)	15 (34.1)	14 (31.8)	>0.999
Previous CABG, *n*	4 (4.5)	3 (6.8)	1 (2.3)	0.616
Peripheral arterial disease, *n*	14 (15.9)	9 (20.5)	5 (11.4)	0.383
Chronic lung disease, *n*	28 (31.8)	16 (36.4)	12 (27.3)	0.493
Previous stroke, *n*	6 (6.8)	2 (4.5)	4 (9.1)	0.676
Malignant tumor, *n*	18 (20.5)	11 (20.5)	7 (15.9)	0.429
Charlson comorbidity index	5.0 ± 1.9	5.6 ± 2.1	4.4 ± 1.5	0.010
NYHA functional class				0.493
I, *n*	60 (68.2)	32 (72.7)	28 (63.6)	
II, *n*	28 (31.8)	12 (27.3)	16 (36.4)	
III/IV, *n*	0 (0.0)	0 (0.0)	0 (0.0)	

Continuous data are presented as means ± standard deviations, except B-type natriuretic peptide (median and interquartile range); categorical data are given as the counts (percentages).

AVR, aortic valve replacement; PCI, percutaneous coronary intervention; CABG, coronary artery bypass grafting; NYHA, New York heart association.

**Table 2:** Echocardiographic findings.

Variables	All patients (*n* = 88)	Medical treatment (*n* = 44, 50.0%)	AVR (*n* = 44, 50.0%)	*p*-value
LVEF, %	58.2 ± 5.2	56.7 ± 5.4	59.6 ± 4.6	0.008
Left ventricular global longitudinal strain, %	−18.1 ± 3.1	−17.2 ± 3.6	−18.9 ± 2.4	0.013
LVEDV index, ml/m^2^	73.1 ± 17.6	77.4 ± 18.6	68.8 ± 15.9	0.022
LVESV index, ml/m^2^	30.9 ± 9.7	33.8 ± 10.2	27.9 ± 8.4	0.004
SV index, ml/m^2^	48.9 ± 7.7	47.4 ± 8.0	50.4 ± 7.2	0.066
Interventricular septal thickness, mm	10.3 ± 1.4	10.5 ± 1.4	10.2 ± 1.5	0.222
Posterior wall thickness, mm	10.1 ± 1.3	10.3 ± 1.2	9.9 ± 1.4	0.165
Left ventricular mass index, g/m^2^	110.2 ± 24.1	113.4 ± 26.5	107.0 ± 21.5	0.218
Relative wall thickness	0.44 ± 0.06	0.44 ± 0.06	0.43 ± 0.07	0.380
Left atrial volume index, ml/m^2^	38.4 ± 22.1	38.9 ± 21.7	37.9 ± 23.0	0.828
Ascending aorta dimension, mm	34.4 ± 4.2	34.4 ± 4.1	34.3 ± 4.4	0.906
Pulmonary artery systolic pressure, mm Hg	30.3 ± 7.9	30.1 ± 9.3	30.6 ± 6.5	0.738
AVA index, cm^2^/m^2^	0.64 ± 0.04	0.65 ± 0.04	0.64 ± 0.04	0.914
Peak AV velocity, m/s	3.40 ± 0.30	3.32 ± 0.25	3.48 ± 0.33	0.015
Peak AVPG, mm Hg	46.6 ± 8.3	44.4 ± 6.9	48.8 ± 9.2	0.013
Mean AVPG, mm Hg	25.6 ± 5.5	24.4 ± 4.9	26.9 ± 5.8	0.033
Velocity ratio	0.32 ± 0.06	0.31 ± 0.06	0.32 ± 0.05	0.293
Bicuspid AV, *n*	11 (12.5)	4 (9.1)	7 (15.9)	0.521
Vena contracta width, mm	4.1 ± 0.7	4.3 ± 0.7	4.0 ± 0.7	0.098
AR jet width/LVOT width, %	35.5 ± 5.6	35.5 ± 4.9	35.3 ± 6.3	0.868
Moderate MR, *n*	4 (4.5)	2 (4.5)	2 (4.5)	>0.999
Moderate MS, *n*	0 (0.0)	0 (0.0)	0 (0.0)	Not applicable
Moderate/severe TR, *n*	8 (9.1)	2 (4.5)	6 (13.6)	0.266

Continuous data are presented as means ± standard deviations; categorical data are given as the counts (percentages).

AVR, aortic valve replacement; LVEF, left ventricular ejection fraction; LVEDV, left ventricular end-diastolic volume; LVESV, left ventricular end-systolic volume; SV, stroke volume; AVA, aortic valve area; AV, aortic valve; AVPG, aortic valve pressure gradient; AR, aortic regurgitation; LVOT, left ventricular outflow tract; MR, mitral regurgitation; MS, mitral stenosis; TR, tricuspid regurgitation.

### Clinical outcomes

**Figure 1:**
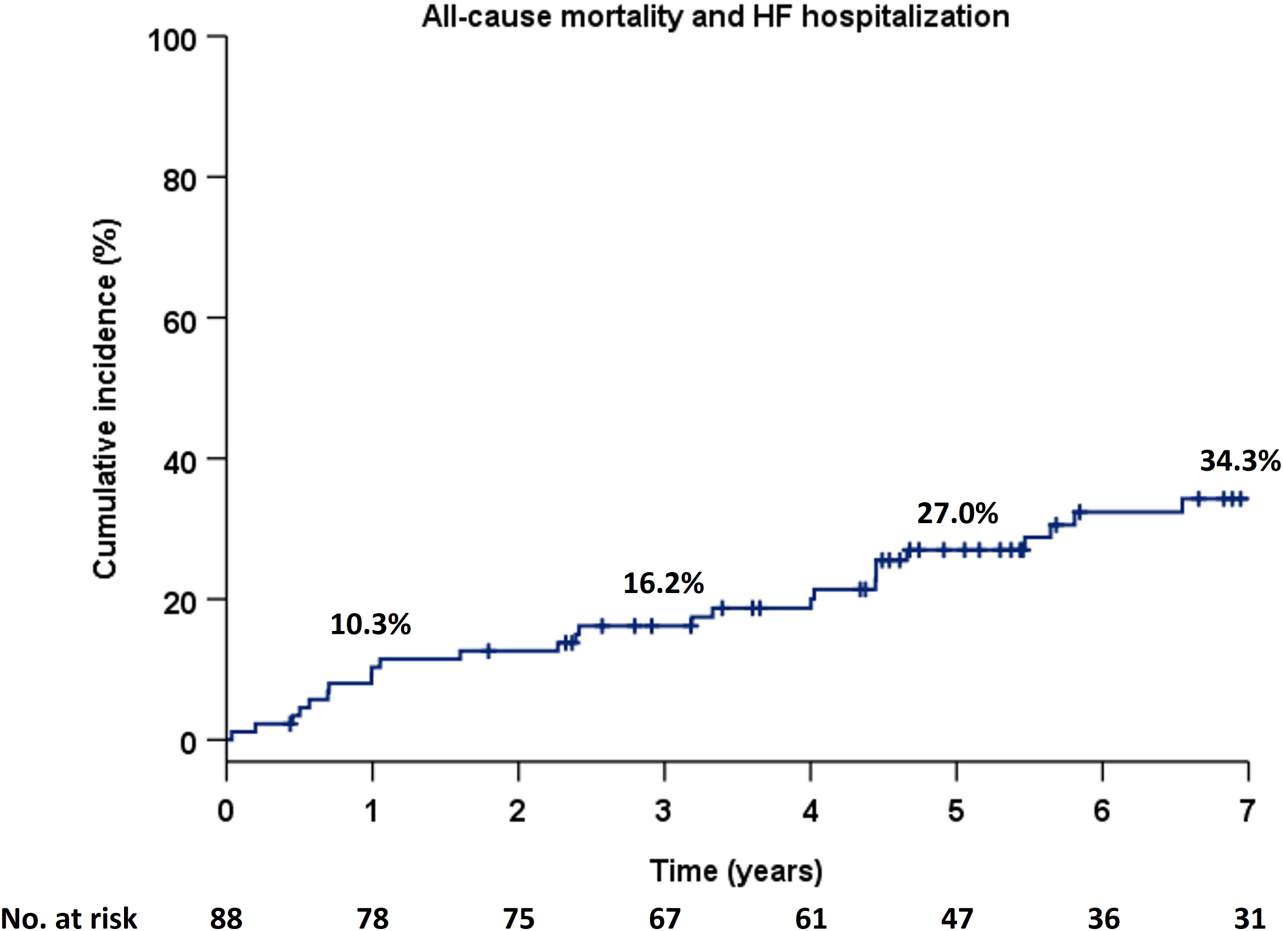
Cumulative incidence of the composite primary endpoint in the Kaplan–Meier method in the total population. HF, heart failure.

The primary endpoint occurred in 32 (36.4%) of the 88 patients during a median follow-up duration of 5.3 years (IQR, 3.2–8.0). No patients with a previous AVR underwent a second AVR during the follow-up period. The Kaplan–Meier estimate for the occurrence of the primary endpoint was 16.2%, 27.0%, and 34.3% at 3, 5, and 7 years, respectively (Figure 1).

### AVR data

AVR was performed in 44 (50.0%) patients during a median follow-up duration of 3.3 years (IQR, 0.5–4.9; Table 3); of these, 23/44 (52.3%) underwent SAVR with the following concomitant procedures, namely, ascending aorta replacement in two (8.7%), CABG in five (21.7%), and ascending aorta replacement with CABG in one (4.3%). TAVR was performed in 21/44 (47.7%) patients with a transfemoral approach used in 18/21 (85.7%) patients. Severity of aortic valve disease at the time of AVR was categorised as moderate MAVD, severe AS, or severe AR in 12/44 (27.2%), 31/44 (70.5%), and 1/44 (2.3%) patients, respectively. Among 12 patients with moderate MAVD, nine patients underwent SAVR and three patients underwent TAVR. In the SAVR patients, isolated SAVR was performed in two patients. Also, three patients underwent concomitant CABG with SAVR. Concomitant ascending aortic replacement, concomitant CABG and ascending aortic replacement, concomitant tricuspid annular repair, and concomitant CABG and tricuspid annular repair with SAVR were performed in one patient each. Isolated SAVR and TAVR were indicated for moderate MAVD mainly because of worsening HF symptoms, progressive severity, and severe calcification of the AV.

**Table 3:** AVR data.

Variables	
AVR, *n*	44/88 (50.0)
SAVR, *n*	23/44 (52.3)
SAVR and ascending aorta replacement, *n*	2/23 (8.7)
SAVR and CABG, *n*	5/23 (21.7)
SAVR, ascending aorta replacement, and CABG, *n*	1/23 (4.3)
TAVR, *n*	21/44 (47.7)
Transfemoral approach, *n*	18/21 (85.7)

Categorical data are given as the counts (percentages).

AVR, aortic valve replacement; SAVR, surgical aortic valve replacement; CABG, coronary artery bypass grafting; TAVR, transcatheter aortic valve replacement.

### Clinical impact of AVR on adverse outcomes

Multivariate Cox regression analysis was used to evaluate AVR as a predictor of the primary endpoint. Age, B-type natriuretic peptide levels, and the Charlson comorbidity index were used for adjustment because the latter two represent extravalvular cardiac damage and comorbidities, respectively (Table 4). AVR was significantly associated with the primary endpoint with a hazard ratio [HR] of 0.248 [95% confidence interval (CI), 0.107–0.579; *p* = 0.001; Table 5]. When the type of AVR, rather than composite of SAVR and TAVR, was used for multivariate Cox regression analysis, both SAVR (HR, 0.146; 95% CI, 0.033–0.650; *p* = 0.012) and TAVR (HR, 0.324; 95% CI, 0.125–0.843; *p* = 0.021) were significantly related to the primary endpoint; here, medical treatment was used as the reference.

**Table 4:** Univariate Cox regression analysis to evaluate predictors for the primary composite endpoint.

Variables	Univariate	
	HR (95% CI)	*p*-value
Age	1.127 (1.047–1.213)	0.001
Men	0.847 (0.420–1.706)	0.641
Chronic kidney disease	3.351 (1.563–7.185)	0.002
B-type natriuretic peptide (per 10 pg/ml increase)	1.035 (1.017–1.053)	<0.001
Atrial fibrillation/flutter	1.257 (0.564–2.799)	0.576
Previous myocardial infarction	2.627 (1.073–6.434)	0.035
Peripheral arterial disease	1.670 (0.678–4.111)	0.265
Chronic lung disease	2.800 (1.395–5.622)	0.004
Previous stroke	1.668 (0.504–5.517)	0.402
Malignant tumor	1.567 (0.700–3.506)	0.275
Charlson comorbidity index	1.751 (1.464–2.093)	<0.001
NYHA functional class II	1.724 (0.849–3.503)	0.132
LVEF	0.933 (0.863–1.008)	0.078
Left ventricular global longitudinal strain	1.160 (1.034–1.301)	0.012
LVEDV index	0.985 (0.965–1.006)	0.168
LVESV index	0.992 (0.955–1.030)	0.678
SV index	0.918 (0.871–0.967)	0.001
Left ventricular mass index	1.018 (1.005–1.032)	0.007
Relative wall thickness (per 0.01 increase)	1.080 (1.026–1.136)	0.003
Left atrial volume index	1.012 (1.000–1.025)	0.054
Pulmonary artery systolic pressure	1.013 (0.967–1.062)	0.577
AVA index (per 0.01 cm^2^/m^2^ increase)	0.927 (0.844–1.018)	0.111
Peak AV velocity	0.552 (0.174–1.751)	0.313
Peak AVPG	0.970 (0.937–1.019)	0.278
Mean AVPG	0.961 (0.898–1.029)	0.254
Velocity ratio (per 0.01 increase)	0.955 (0.895–1.018)	0.156
Bicuspid AV	0.546 (0.130–2.294)	0.410
Vena contracta width	1.412 (0.889–2.242)	0.144
AR jet width/LVOT width	1.024 (0.962–1.091)	0.453
Moderate MR	2.777 (0.842–9.152)	0.093
Moderate TR	1.487 (0.521–4.248)	0.459
AVR	0.207 (0.092–0.466)	<0.001
Type of AVR		
Medical treatment	Reference	
SAVR	0.088 (0.021–0.377)	0.001
TAVR	0.369 (0.150–0.908)	0.030
Concomitant CABG	0.435 (0.059–3.199)	0.414

HR, hazard ratio; CI, confidence interval; NYHA, New York heart association; LVEF, left ventricular ejection fraction; LVEDV, left ventricular end-diastolic volume; LVESV, left ventricular end-systolic volume; SV, stroke volume; AVA, aortic valve area; AV, aortic valve; AVPG, aortic valve pressure gradient; AR, aortic regurgitation; LVOT, left ventricular outflow tract; MR, mitral regurgitation; TR, tricuspid regurgitation; AVR, aortic valve replacement; SAVR, surgical aortic valve replacement; TAVR, transcatheter aortic valve replacement; CABG, coronary artery bypass grafting.

**Table 5:** Multivariate Cox regression analysis to evaluate predictors for the primary composite endpoint.

Variables	HR (95% CI)	*p*-value
AVR	0.248 (0.107–0.579)	0.001
Type of AVR		
Medical treatment	Reference	
SAVR	0.146 (0.033–0.650)	0.012
TAVR	0.324 (0.125–0.843)	0.021

Age, Charlson comorbidity index, and B-type natriuretic peptide were included as adjustment factors.

HR, hazard ratio; CI, confidence interval; AVR, aortic valve replacement; SAVR, surgical aortic valve replacement; TAVR, transcatheter aortic valve replacement.

**Figure 2:**
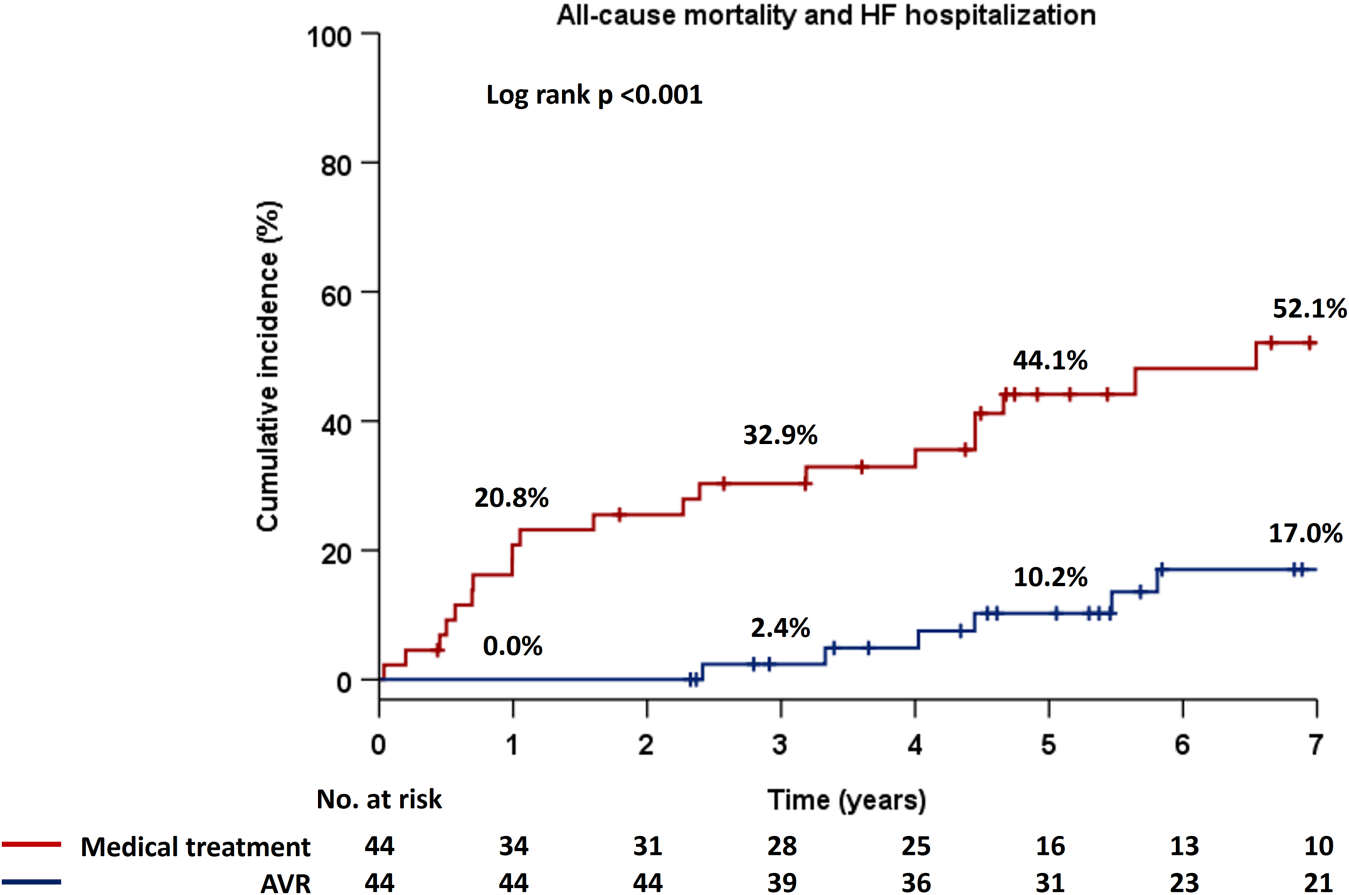
Cumulative incidence of the composite primary endpoint in the Kaplan–Meier method in two groups, i.e., medical treatment and AVR. AVR, aortic valve replacement; HF, heart failure.

**Figure 3:**
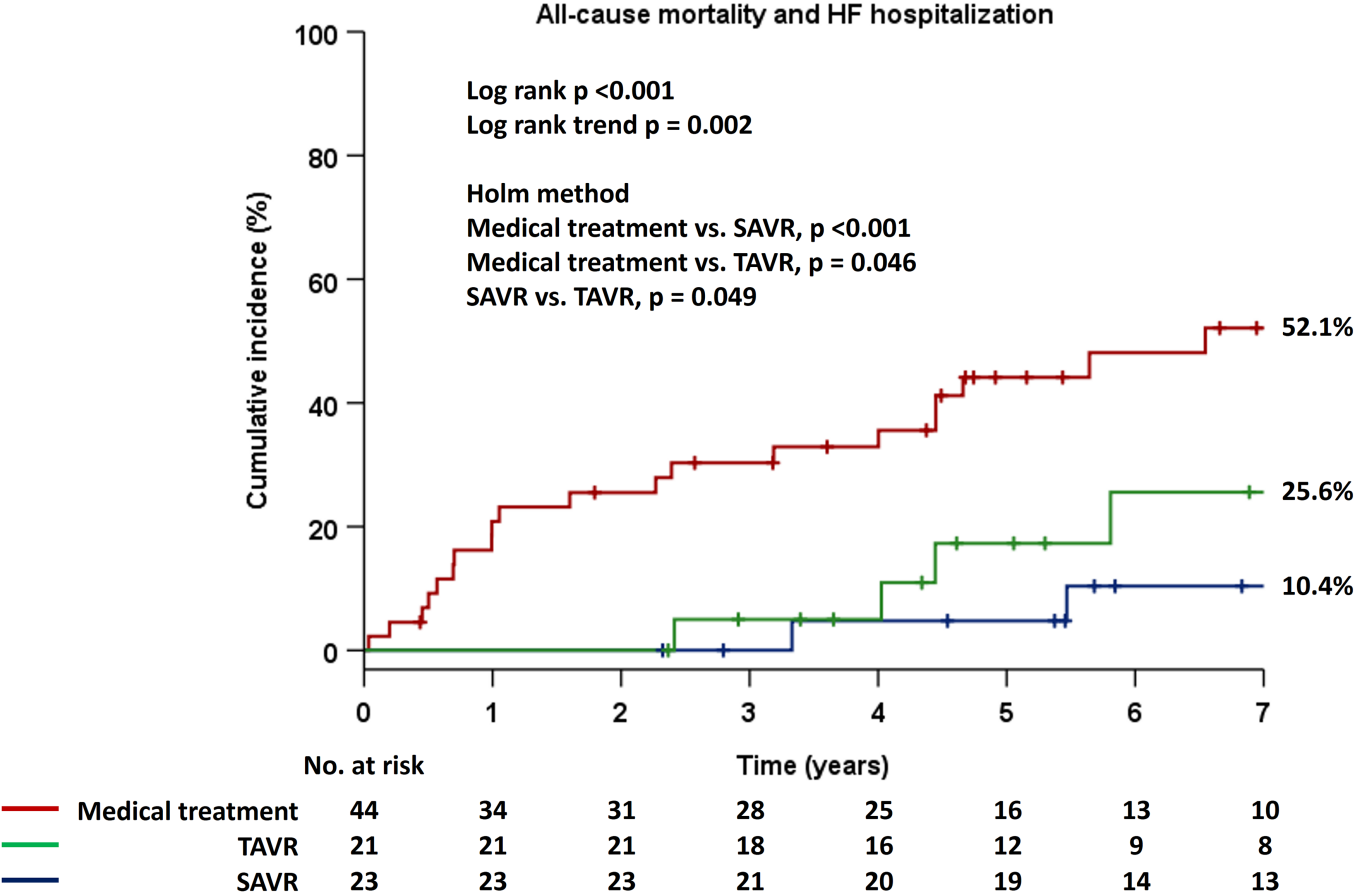
Cumulative incidence of the composite primary endpoint in the Kaplan–Meier method in the three groups, i.e., medical treatment, TAVR, and SAVR. AVR, aortic valve replacement; HF, heart failure; SAVR, surgical aortic valve replacement; TAVR, transcatheter aortic valve replacement.

Patients were stratified into two groups based on management strategy as medical treatment or AVR, and Kaplan–Meier estimates for the primary endpoint demonstrated significantly higher event rates in patients with medical treatment compard to those who had undergone AVR (*p* < 0.001) (Figure 2). Notably, there were no adverse events in the AVR group for the first two years. Next, patients were divided into three groups based on the types of AVR, i.e., medical treatment, SAVR, and TAVR, and there were significant differences in the incidence rates of the primary endpoint among the three groups (*p* < 0.001) (Figure 3). Interstingly, the Holm method revealed that patients with SAVR had significantly better outcomes compared to those provided medical treatment (*p* < 0.001) or TAVR (*p* = 0.049), and similarly, there were significant differences between patients provided medical treatment and TAVR (*p* = 0.046).

### Comparison of patient characteristics between the medical treatment and AVR groups

The results of the comparison of patient data among medical treatment and AVR groups are shown in Tables 1, 2, and significantly lower rates of diabetes mellitus and previous myocardial infarction, along with a relatively lower rate of chronic kidney disease, was seen in patients with AVR. Additionally, patients with AVR had significantly lower Charlson comorbidity index scores than those managed with medical treatment. Next, compared to those managed with medical treatment, patients with AVR had significantly better left ventricular parameters, including LVEF, left ventricular global longitudinal strain, and left ventricular end-diastolic and end-systolic volume indices but worse AV hemodynamic parameters, such as peak AV velocity and peak and mean AVPG.

## Discussion

This retrospective analysis among moderate MAVD patients revealed that 50% of the patients underwent AVR during the follow-up period and that AVR might be associated with favorable clinical outcomes, irrespective of the type of AVR performed.

### Moderate MAVD as a progressive disease and AVR during the follow-up period

We have previously reported that the cumulative incidence of AVR was 40.9% at 5 years and that this value was higher than that of all-cause mortality and HF hospitalization (7). At least one other study has reported a similarly high cumulative incidence for AVR, i.e., 65% at 5 years (6). In our cohort, half of the patients with moderate MAVD underwent AVR during the follow-up period, indicating that AVR might eventually be required in such patients, probably because of the potential for extravalvular cardiac damage. Additionally, MAVD is a progressive condition because it has been reported that medical management of the disease led to peak AV velocity increase, LVEF decrease, and significant LV size enlargement during one-year of follow-up (7). Thus, AVR may retard the deterioration in AV hemodynamics and ameliorate clinical outcomes; nevertheless, this aspect represents challenge to the clinical relevance of AVR in patients with moderate MAVD.

### Potential impact of AVR on clinical outcomes

Previous studies have demonstrated that AVR is associated with favorable outcomes in patients with moderate-to-severe MAVD (2, 3); however, among such patients with reduced LVEF pre-AVR but high left ventricular mass index and relative wall thickness, AVR led to a greater number of cardiovascular adverse events [ischemic stroke, HF hospitalization, severe left ventricular dysfunction (LVEF <35%), or cardiac death] compared to those with low left ventricular mass index, low relative wall thickness, and high LVEF pre-AVR. Further, this effect was more pronounced among patients with higher left ventricular mass index and relative wall thickness compared to those with reduced LVEF (16), suggesting that AVR for moderate-to-severe MAVD should be considered before extravalvular cardiac damage worsens.

While patients with moderate AS have unfavorable clinical outcomes (4, 5), there are currently two ongoing randomized trials investigating TAVR for moderate AS. These are the PROGRESS (A Prospective, Randomized, Controlled Trial to Assess the Management of Moderate Aortic Stenosis by Clinical Surveillance or Transcatheter Aortic Valve Replacement) trial (NCT04889872), and the Evolut™ EXPAND TAVR II Pivotal Trial (NCT05149755), which are expected to provide insights into whether early TAVR could offer safety and efficacy for such patients. There is paucity of data on the clinical relevance of AVR in patients with moderate MAVD, and to the best of our knowledge, this is first report to suggest that AVR on-demand might be associated with favorable clinical outcomes in such patients because, notably, patients in our cohort who underwent AVR did not experience any primary endpoint events during the first two years post-procedure. Further, previous studies have described that high relative wall thickness and BNP levels in moderate MAVD, which indicate the presence of extravalvular cardiac damage, are associated with adverse clinical outcomes (6, 7). Given that moderate MAVD is a progressive condition (7), such patients should be followed-up carefully, with indications for AVR meticulously discussed if they develop symptoms of HF, or display worsening AV hemodynamics and progressive extravalvular cardiac damage during follow-up.

## Conclusions

As about half of the patients with moderate MAVD and preserved LVEF eventually underwent AVR during the follow-up period, and AVR may be associated with favourable clinical outcomes in such patients. Careful follow-up of these patients and monitoring for indications of AVR during the follow-up period are needed.

## Study limitations

This study is subject to certain constraints. Firstly, it is a retrospective examination of a modest patient cohort, which could potentially introduce bias in the data gathering process. As such, these outcomes should be corroborated by larger-scale, multicenter prospective studies. Secondly, in contrast to prior studies, the severity of AR was ascertained using semi-quantitative and qualitative methodologies, rather than a quantitative or integrated approach incorporating the proximal isovelocity surface area method, inclusive of parameters such as effective regurgitant orifice area, regurgitant volume, and regurgitant fraction (6, 17). Despite these limitations, a previous study indicated a significant correlation between the vena contracta width and such quantitative indices, regardless of the presence of central or eccentric AR jets (18). Thus, it was considered justified to use the vena contracta width for AR grade evaluation. Lastly, both categories of AVR, i.e., SAVR, and TAVR, were significantly linked to positive clinical outcomes; however, the incidence of the primary endpoint was somewhat elevated in patients who underwent TAVR, a finding that could potentially be ascribed to a type I error.

## Data Availability

The raw data supporting the conclusions of this article will be made available by the authors, without undue reservation.

## References

[1] Ngiam JN Chew NWS Pramotedham T Tan BY Sia CH Loh PH Implications of coexisting aortic regurgitation in patients with aortic stenosis. JACC Asia. (2021) 1:105–11. 10.1016/j.jacasi.2021.05.004 36338366 PMC9627873

[2] Isaza N Desai MY Kapadia SR Krishnaswamy A Rodriguez LL Grimm RA Long-term outcomes in patients with mixed aortic valve disease and preserved left ventricular ejection fraction. J Am Heart Assoc. (2020) 9(7):e014591. 10.1161/JAHA.119.014591 32204665 PMC7428636

[3] Saijo Y Isaza N Conic JZ Desai MY Johnston D Roselli EE Left ventricular longitudinal strain in characterization and outcome assessment of mixed aortic valve disease phenotypes. JACC Cardiovasc Imaging. (2021) 14:1324–34. 10.1016/j.jcmg.2021.01.020 33744141

[4] Strange G Stewart S Celermajer D Prior D Scalia GM Marwick T Poor long-term survival in patients with moderate aortic stenosis. J Am Coll Cardiol. (2019) 74:1851–63. 10.1016/j.jacc.2019.08.004 31491546

[5] Delesalle G Bohbot Y Rusinaru D Delpierre Q Marechaux S Tribouilloy C. Characteristics and prognosis of patients with moderate aortic stenosis and preserved left ventricular ejection fraction. J Am Heart Assoc. (2019) 8:e011036. 10.1161/JAHA.118.011036 30841771 PMC6475062

[6] Egbe AC Luis SA Padang R Warnes CA. Outcomes in moderate mixed aortic valve disease: is it time for a paradigm shift? J Am Coll Cardiol. (2016) 67:2321–9. 10.1016/j.jacc.2016.03.509 27199054

[7] Onishi H Naganuma T Izumo M Ouchi T Yuki H Mitomo S Prognostic relevance of B-type natriuretic peptide in patients with moderate mixed aortic valve disease. ESC Heart Fail. (2022). 10.1002/ehf2.13946 35543340 PMC9288736

[8] Baumgartner H Hung J Bermejo J Chambers JB Edvardsen T Goldstein S Recommendations on the echocardiographic assessment of aortic valve stenosis: a focused update from the European association of cardiovascular imaging and the American society of echocardiography. J Am Soc Echocardiogr. (2017) 30:372–92. 10.1016/j.echo.2017.02.009 28385280

[9] Zoghbi WA Adams D Bonow RO Enriquez-Sarano M Foster E Grayburn PA Recommendations for noninvasive evaluation of native valvular regurgitation: a report from the American society of echocardiography developed in collaboration with the society for cardiovascular magnetic resonance. J Am Soc Echocardiogr. (2017) 30:303–71. 10.1016/j.echo.2017.01.007 28314623

[10] Charlson ME Pompei P Ales KL MacKenzie CR. A new method of classifying prognostic comorbidity in longitudinal studies: development and validation. J Chronic Dis. (1987) 40:373–83. 10.1016/0021-9681(87)90171-8 3558716

[11] Baumgartner H Hung J Bermejo J Chambers JB Evangelista A Griffin BP Echocardiographic assessment of valve stenosis: EAE/ASE recommendations for clinical practice. J Am Soc Echocardiogr. (2009) 22:1–23; quiz 101–102. 10.1016/j.echo.2008.11.029 19130998

[12] Mitchell C Rahko PS Blauwet LA Canaday B Finstuen JA Foster MC Guidelines for performing a comprehensive transthoracic echocardiographic examination in adults: recommendations from the American society of echocardiography. J Am Soc Echocardiogr. (2019) 32:1–64. 10.1016/j.echo.2018.06.004 30282592

[13] Otto CM Nishimura RA Bonow RO Carabello BA Erwin JP 3rd Gentile F 2020 ACC/AHA guideline for the management of patients with valvular heart disease: a report of the American college of cardiology/American heart association joint committee on clinical practice guidelines. J Am Coll Cardiol. (2021) 77:e25–e197. 10.1016/j.jacc.2020.11.018 33342586

[14] Cepeda MS Boston R Farrar JT Strom BL. Comparison of logistic regression versus propensity score when the number of events is low and there are multiple confounders. Am J Epidemiol. (2003) 158:280–7. 10.1093/aje/kwg115 12882951

[15] Peduzzi P Concato J Kemper E Holford TR Feinstein AR. A simulation study of the number of events per variable in logistic regression analysis. J Clin Epidemiol. (1996) 49(12):1373–9. 10.1016/s0895-4356(96)00236-3 8970487

[16] Egbe AC Warnes CA. Cardiovascular adverse events after aortic valve replacement in mixed aortic valve disease: beyond ejection fraction. J Am Coll Cardiol. (2016) 68:2591–3. 10.1016/j.jacc.2016.09.948 27931620

[17] Rashedi N Popovic ZB Stewart WJ Marwick T. Outcomes of asymptomatic adults with combined aortic stenosis and regurgitation. J Am Soc Echocardiogr. (2014) 27:829–37. 10.1016/j.echo.2014.04.013 24874975

[18] Tribouilloy CM Enriquez-Sarano M Bailey KR Seward JB Tajik AJ. Assessment of severity of aortic regurgitation using the width of the vena contracta: a clinical color Doppler imaging study. Circulation. (2000) 102(5):558–64. 10.1161/01.cir.102.5.558 10920069

